# Bone marrow cellular profile in patients with diabetes: association of statin therapy with CD34+ cells

**DOI:** 10.3389/fendo.2026.1850519

**Published:** 2026-05-29

**Authors:** Dominika Sojakova, Tetiana Osadcha, Ludek Horvath, Jitka Husakova, Vladimíra Fejfarova, Karol Sutoris, Radka Jarosikova, Michal Dubsky

**Affiliations:** 1 Diabetes Centre, Institute for Clinical and Experimental Medicine, Videnska, Prague, Czechia; 2 First Faculty of Medicine Charles University, Katerinska, Prague, Czechia; 3 Department of Data Science, Institute for Clinical and Experimental Medicine, Videnska, Prague, Czechia; 4 Transplantation Surgery Department, Institute for Clinical and Experimental Medicine, Videnska, Prague, Czechia

**Keywords:** bone marrow, diabetes, peripheral artery disease, statins, stem cells

## Abstract

**Backgrounds:**

Diabetes is associated with alterations in bone marrow (BM) cellularity beyond its acute and chronic complications in both type 1 and type 2 diabetes. These changes may be amplified in patients with advanced disease, such as CLTI. Key alterations include imbalance of the BM microenvironment and shifts in hematopoietic cell populations, particularly the CD34+ subpopulation.

**Methods:**

This retrospective observational study analyzed patients treated with autologous cell therapy (ACT) for chronic limb-threatening ischemia over 16 years. Patients on immunosuppressive therapy or corticoids, with stage 4–5 chronic kidney disease, repeated ACT were excluded. Harvested BM underwent hematological analysis (leucocytes, monocytes, lymphocytes) and immunophenotyping (percentage and absolute count of CD34). Outcomes were evaluated in relation to diabetes parameters (type, duration, glycated hemoglobin, diabetic complications score index - DCSI) and medications (oral antidiabetics, statins). Wilcoxon rank test and Spearman correlation were used.

**Results:**

Seventy-four patients were included from 167 in the ACT database. Statin therapy was significantly associated with higher CD34 percentage (β = 0.256, p< 0.001), while simultaneously being associated with lower lymphocyte counts, suggesting that statins may modify BM cellular composition rather than directly increase absolute progenitor cell numbers.

**Conclusion:**

Statins appear to optimize the BM microenvironment rather than increase absolute hematopoietic cell numbers in a non-hematological diabetic population with severe limb ischemia. In addition, higher DCSI scores were associated with increased CD34+ cell percentage, suggesting a link between diabetes severity and BM cellular composition.

## 1 Introduction

Diabetes mellitus (DM) is a chronic disease projected to affect 588.7 million people worldwide by 2024, with its incidence steadily rising (1). In addition to well-established microvascular and cardiovascular complications, bone marrow (BM) function is impaired in patients with diabetes due to both vascular and cellular alterations (2, 3). The mechanisms underlying BM dysfunction differ between type 1 (T1D) and type 2 diabetes (T2D). In T1D, key contributors include autonomic neuropathy and neurohormonal dysregulation, which result in BM microangiopathy, hypoperfusion, a decline in progenitor cells, and impaired mobilization (2, 4, 5). In contrast, BM dysfunction in T2D is primarily driven by adipose tissue remodeling, the accumulation of advanced glycation end products (AGEs), and chronic inflammation (6–8). These changes lead to endothelial dysfunction, reduced vascular conductance, and a decrease in osteoprogenitor mesenchymal stromal cell populations (3, 9). Furthermore, patients with peripheral arterial disease (PAD) exhibit pronounced BM adipogenesis and microangiopathy, characterized by damage to BM sinusoids and hypoperfusion. As a result, hematopoietic cellularity is further reduced in individuals with T2D and coexisting PAD compared to those with T2D alone (2, 10).

Nevertheless, glycemic control also influences BM cellular quality and function. Patients with poorer glycemic control have significantly lower absolute levels of CD34+ stem/progenitor cells compared to individuals with better glycemic status (11). Chronic exposure to hyperglycemia induces persistent epigenetic and functional defects in CD34 cells, further reducing their number and regenerative potential, even if normoglycemia is subsequently restored (12). On the other hand, acute hypoglycemia also abolishes the normal diurnal decline of endothelial progenitor cells (EPC) and reduces CD34 stem cells during severe hypoglycemic episodes (13).

Another issue regarding BM changes is their relationship to oral antidiabetic drugs (OADs), angiotensin-converting enzyme inhibitors (ACEI), and statins. In this study, we focused on commonly prescribed medications in patients with diabetes and no-option CLTI that have documented effects on BM biology. The main effect of statins in BM is to increase the mobilization and activity of specific types of stem cells and to promote a regenerative and less inflammatory BM environment (14–17). Metformin improves the immune and oxidative stress environment of the BM, primarily by modifying immune cell crosstalk (particularly with macrophages) and suppressing chronic inflammation, thereby promoting a healthier environment for hematopoiesis and osteogenesis (18, 19). This raises questions about the impact of newer OADs, like sodium-glucose cotransporter-2 (SGLT-2) inhibitors, on BM cellularity. ACEI therapy has been shown to modulate bone-marrow cellularity by suppressing local renin–angiotensin signaling within the marrow niche, which may lead to alterations in progenitor-cell numbers and erythroid lineage activity (20, 21). This study aimed to characterize BM cellular composition in patients with diabetes undergoing autologous cell therapy (ACT) and to explore its associations with selected clinical parameters, including diabetes-related factors, comorbidities, and concomitant medication.

## 2 Materials and methods

### 2.1 Study population

We analyzed data from patients who were indicated to undergo autologous cell therapy (ACT), a treatment option for patients with diabetes and chronic limb-threatening ischemia (CLTI), who lack the possibility of standard revascularization. Therefore, the study inclusion criteria were diagnosis of type 1 or type 2 diabetes with a duration of ≥3 years; age 18–90 years; diabetic foot (an ulcer distal from ankle) and/or status after minor amputation, in accordance with international classification TEXAS 2-3 (C-D), Wagner 2-4; presence of CLTI - ischemic ulceration, gangrene or rest pain lasting ≥2 weeks; transcutaneous oxygen pressure (TcPO_2_) <35 mmHg, and non-eligibility for standard revascularization (PTA or by-pass). Exclusion criteria were the presence of general signs of severe infection, deep foot infection with phlegmon, severe edema of the limb, severe hematological abnormalities, deep vein thrombosis, myocardial infarction or stroke in the last 6 months, untreated severe diabetic retinopathy, a diagnosed malignancy, pregnancy, breastfeeding, and inability to undergo general anesthesia.

After the assessment of all inclusion and exclusion criteria, eligible patients underwent standard blood tests (full blood count, C-reactive protein (CRP), creatinine, and standard oncological screening (chest X-ray, abdominal ultrasound, tumor markers, fecal occult blood test, PSA level in men, and mammogram in women) as well as ophthalmological fundus examination. If there were no contraindications after these examinations, we proceeded with pre-screening, which included X-rays of the affected limb; serological tests (hepatitis panel, HIV, and syphilis testing); coagulation testing; complete blood count; biochemical parameters (renal and liver functional parameters, mineral analysis, albumin, lipid profile and Hb1Ac); and C-reactive protein.

**Figure 1:**
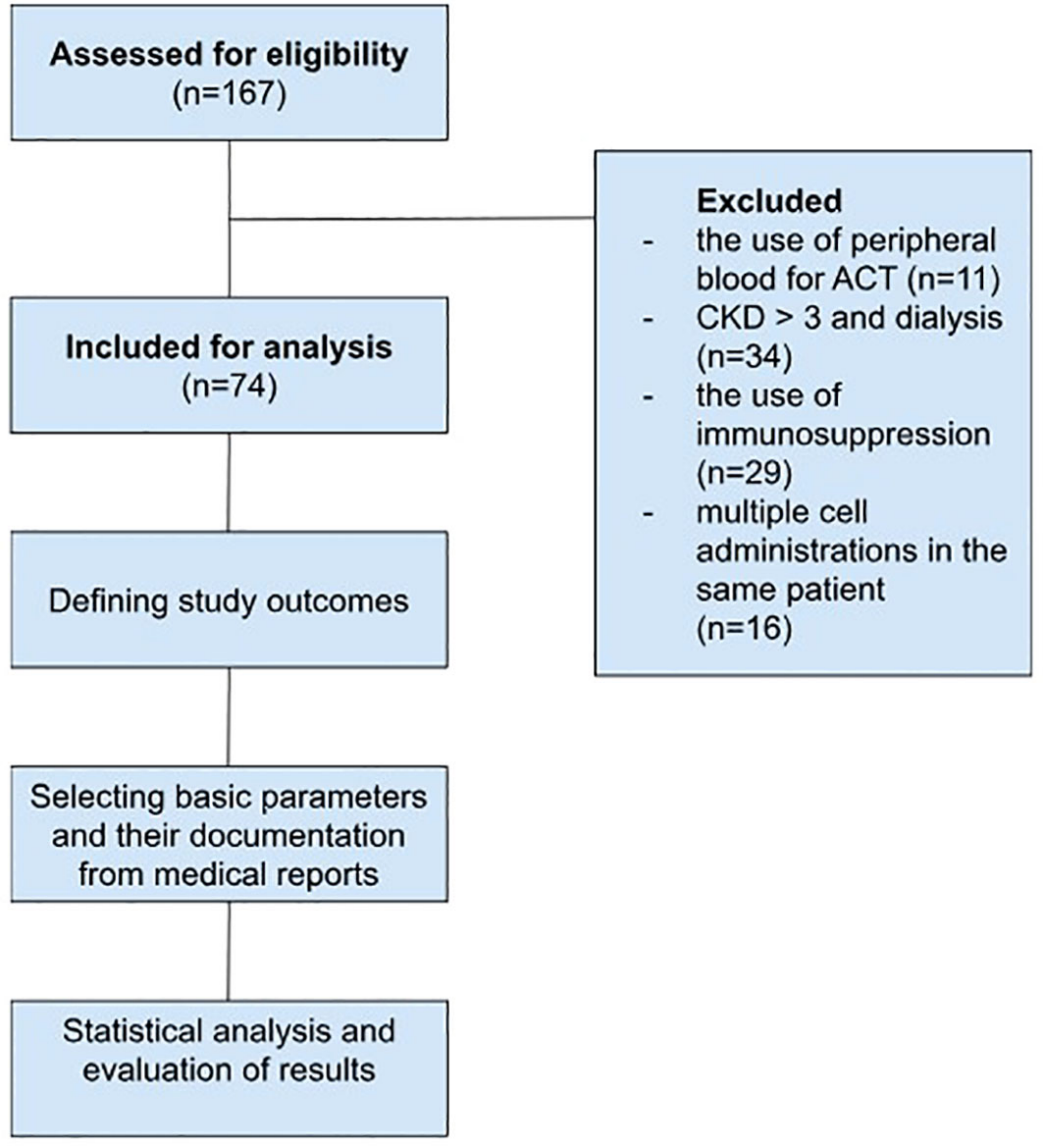
Flowchart of retrospective patient selection. ACT, autologous cell therapy; CKD, chronic kidney disease.

For this particular analysis, we excluded data from repeated administration of ACT, thus utilizing only the results of the first treatment, and excluded patients in whom stimulated peripheral blood was used instead of BM. Furthermore, we excluded patients with advanced chronic kidney disease (stage G4 and G5), including dialysis patients, due to changes induced by renal impairment and the regular use of erythropoietin and internal environment-modifying supplements (22, 23). Finally, we decided not to include patients taking any type of immunosuppressants, because of its potential effects on BM function. The flowchart showing patient selection is shown in **Figure 1**.

### 2.2 BM harvest and immunophenotype analysis

The patients were hospitalized at the Diabetes center of the Institute for Clinical and Experimental Medicine for an average of 5 days, and the procedure was performed under antibiotic prophylaxis and anesthesia, as determined by the anesthesiologist and patient preference (epidural, spinal or general anesthesia). The patient was transferred to the operating theatre and placed in a lateral position, then a surgeon harvested 250ml of BM using a Jamshidi needle and BM collection bag (Compoflex 1F – Empty – 600 – Luer Male – Seg. Tubes). After that, the harvested BM was mixed with 5 mL of heparin solution and acid–citrate–dextrose solution. Then a sample was taken from the total volume for hematological and immunophenotypic analysis into two 2-mL EDTA tubes, which were used for subsequent analyses.

CD34 hematopoietic progenitor cells were quantified using flow cytometry (Beckman Coulter, Navios EX) in accordance with standardized protocols. Cells were stained with fluorochrome-conjugated monoclonal antibodies against CD34 and CD45, and viability was assessed using 7-AAD or an equivalent viability dye. The gating strategy followed the ISHAGE (International Society of Hematotherapy and Graft Engineering) guidelines, involving sequential gating to exclude debris, doublets, and non-viable cells, with identification of CD45^dim/CD34^bright events within the lymphocyte/mononuclear region (24). BM aspirates were also subjected to standard hematological analysis. Complete blood count (CBC) parameters were assessed using an automated hematology analyzer (Sysmex XN-10 and XN-20). The analysis included quantification of total leukocytes, erythrocytes, thrombocytes, hemoglobin, and hematocrit, as well as calculation of red cell indices (MCV, MCH, MCHC) and platelet indices (MPV, PDW).

The studies involving human participants were reviewed and approved by the ethics committee of the Institute for Clinical and Experimental Medicine (IKEM) and Thomayer University Hospital. The patients/participants provided their written informed consent before participation in the study.

### 2.3 Study schedule

All data were retrospectively collected during the admission examination at the department, i.e., 1–2 days before cell therapy. In this study, we focused on demographic information (age and sex), parameters of diabetes (type, duration and HbA1c) and the lipid profile (LDL and non-HDL). Except for the stage of chronic kidney disease (CKD), we assessed complications of diabetes following the diabetes complication severity index (DCSI). It provides a standardized, reproducible measure of the overall burden of diabetes complications. DCSI includes seven categories of diabetes-related complications - cardiovascular disease, nephropathy, retinopathy, peripheral arterial disease, stroke, neuropathy, and metabolic complications (severe hypoglycemia and ketoacidosis). Neuropathy was assessed as the presence of diabetic peripheral or autonomic neuropathy, defined on the basis of clinical manifestations and neurological examination, including impaired monofilament sensation (inability to perceive all three test applications) and/or reduced vibration perception measured by biothesiometry (> 15V). The DCSI classifies neuropathy as an irreversible chronic complication associated with impaired neurovascular function, loss of protective sensation, and an increased risk of ulceration and amputation. The maximal total DCSI score is 13 points. Higher DCSI scores are associated with an increased risk of death, more frequent hospitalizations, and greater health care costs (25). Finally, we correlated statins and OADs used by patients in this study, including insulin, metformin, dipeptidyl peptidase 4 (DPP-4) inhibitors and SGLT-2 inhibitors, with BM parameters. Although glucagon-like peptide-1 (GLP-1) agonists belong to the newer antidiabetic drugs, only a small number of patients in our cohort used them, and therefore they were not included into this analysis.

The number of CD34 subpopulation was selected as a primary outcome because it represents a classical marker of hematopoietic cells, particularly hematopoietic stem and progenitor cells, both in research and clinical transplantation (26, 27). For the assessment of the entire BM microenvironment, the percentage of CD34 (%CD34) was chosen. The number of mononuclear cells, Additional outcomes included lymphocytes and monocytes, and the monocyte-to-lymphocyte ratio (MLR), reflecting the immunological and supportive environment of the BM.

### 2.4 Statistical analysis

To identify significant predictors of the outcome variable, a two-step approach was employed. First, we conducted a univariate analysis to test the relationship between each predictor and the outcome. Univariate associations were assessed using nonparametric methods (Wilcoxon rank-sum test and Spearman correlation) due to the non-normality of the outcome. Variables demonstrating a statistically significant association with the outcome in the univariate analyses were later included in the multivariate regression model to evaluate their independent effects. In addition, age, sex, and duration of diabetes mellitus were included in all multivariate models regardless of their univariate significance, based on their clinical relevance. The duration of diabetes mellitus was included in models that included diabetes type as a predictor. For multivariable modelling, the outcome was transformed using the Box-Cox transformation to approximate normality and stabilize variance.

## 3 Results

### 3.1 Baseline patient characteristics

We included 74 patients in this study, 10 females and 64 males, a mean age of 69.0 years, as is summarized in **Table 1**, together with all baseline characteristics. The majority of the patients were diagnosed with T2D (65/74 patients) with a duration of approximately 21 years. The interquartile range of HbA1c (59.0 mmol/mol) reflects their poor glycemic control. DCSI showed that most of them scored 7 out of 13 points, with three-quarters diagnosed with diabetic neuropathy (73%), more than half with nephropathy (56.8%) and coronary artery disease (62.2%) and approximately one-third with retinopathy (37.8%).

**Table 1:** Baseline characteristics of the patients, complications and medication.

Baseline parameters	Stage	Value
Sex female		10/74 (13.5%)
Age (years)		69.0 (64.0 – 75.0)
Active smokers		10/74 (13.5%)
Type 1 diabetes		9/74 (12.2%)
Type 2 diabetes		65/74 (87.8%)
Duration [years]		21.0 (15.0 – 29.8)
HbA1c [mmol/mol]		59.0 (52.0 – 69.0)
Complications		
Chronic kidney disease	2	23/74 (31.1%)
	3	19/74 (25.7%)
Diabetic retinopathy	Non-proliferative	15/74 (20.3%)
	Proliferative	13/74 (17.6%)
Diabetic neuropathy		54/74 (73%)
Coronary artery disease		46/74 (62.2%)
DCSI score	1 - 5	26/74 (35.1%)
	6 - 9	45/74 (60.8%)
	10 - 13	2/74 (2.7%)
Risk factors		
Smoking		10/74 (13.5%)
Total cholesterol [mmol/L]		4.0 (3.4 – 4.5)
LDL [mmol/L]		2.2 (1.7–2.9)
HDL [mmol/L]		0.9 (0.8 – 1.2)
non-HDL [mmol/L]		2.9 (2.2–3.5)
Medication		
Insulin		54/74 (73.0%)
Metformin		24/74 (32.4%)
DPP4i		9/74 (12,2%)
SGLT2i		7/74 (9.5%)
Statins		48/74 (64.7%)
Atorvastatin		31/74 (41.9%)
Rosuvastatin		10/74 (13.5%)
Simvastatin		5/74 (6.8%)
Fluvastatin		2/74 (2.7%)

DCSI, diabetes complications severity index; DPP4i, dipeptidyl peptidase-4 inhibitors; SGLT2i, sodium-glucose cotransporter 2 inhibitors. All variables are expressed by number (percentage) or median (interquartile range 25% - 75%).

Regarding antidiabetic treatment, 73% of patients were treated with insulin and 32% with metformin. Only 12% of patients were treated with DPP-4 inhibitors and 9.5% with SGLT-2 inhibitors. Since other groups of antidiabetic drugs, including GLP-1 analogues, were used only rarely in our patient group, they were not included as variables in this study. In addition to diabetes therapy, we assessed ACEI and statin use, which was present in 64.9% of patients, mostly atorvastatin (41.9%). The majority of patients in this cohort were not followed in specialized diabetology outpatient care at our center.

### 3.2 BM cellular composition

After hematological assessment and flow cytometry analysis, we summarized the cellular parameters, which are shown in **Table 2**. From these parameters, we selected the predictors with the highest explanatory value for describing BM cellularity. Univariate analysis was performed on each outcome variable. As the dataset was relatively small, we did not include all variables at once in a single multivariate model, and only variables that were statistically significant (p < 0.05) in the univariate analysis were included in the multivariate analysis, together with age, sex, and duration of diabetes.

**Table 2:** Number of monitored elements in bone marrow.

Parameter	Value
CD34 percentage	0.6 (0.4 – 0.8) %
CD34 absolute count	53.3 (27.5 – 88.0) 10^6/l
Leucocytes	9.9 (I8.0 – 12.9) 10^9
Monocytes	0.5 (0.2 – 0.7) 10^9
Lymphocytes	1.8 (0.9 – 3.3) 10^9
Thrombocytes	101.0 (50.0 – 153.8) 10^9
MPV	10.1 (7.9 – 11.5) fl
Viability	98.0 (96.6–99.0) %

Data are expressed by median (interquartile range - IQR), MPV, mean platelet volume.

### 3.3 Statins and CD34 parameters

Patients receiving statin therapy had higher %CD34 values compared with patients without statin therapy (median 0.67 vs. 0.43, W=293, p = 0.0004), and this association remained highly significant after adjustment for age and sex in multivariate analysis (β = 0.256, p < 0.001). Statin therapy was associated with significantly higher %CD34 in males (0.671 vs. 0.411; p < 0.001) and in patients with T2D (0.619 vs. 0.414; p = 0.003). No significant association was observed in females or patients with T1D. Stratification according to statin dose or category did not reveal any significant differences. Given the relatively small representation of females (n= 5 vs 5 patients) and patients with type 1 diabetes (n= 2 vs 7 patients), these findings should be interpreted with caution.

**Figure 2:**
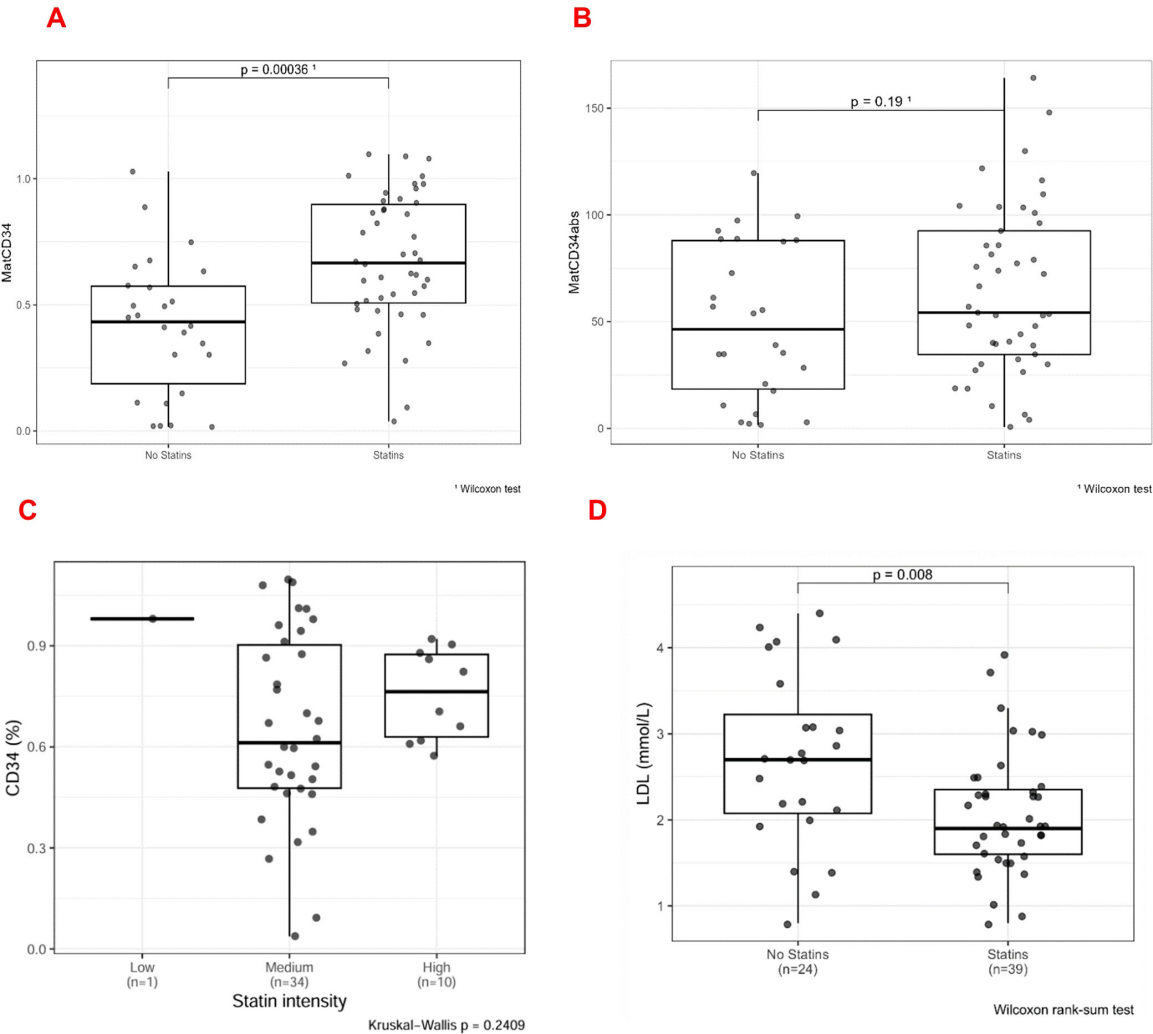
The proportion of CD34 was significantly associated with statin therapy **(A)**, whereas the absolute CD34 cell count was not significantly influenced by statin use **(B)**. No correlation was observed between LDL levels and %CD34 **(C)**. Stratification according to statin intensity classification did not reveal significant differences in %CD34 between groups **(D)**.

There was no correlation between the value of LDL and % CD34 (β = -0.006, p= 0.89) and a significant association between LDL and statin therapy (p= 0.008). Statin doses were evaluated according to statin intensity classification (low-, moderate-, and high-intensity), and there was no significant difference between groups (p = 0.55) (28). Statin therapy also did not influence MLR (median -1.68 vs. -1.00, W=457.5, p = 0.10). An overview of these results is provided in **Figure 2**.

To explain the discrepancy between relative and absolute CD34 counts, additional analyses evaluating the possible reduction of other BM cell populations were performed. In our cohort, lymphocyte count was the only parameter that showed a statistically significant association consistent with this interpretation (median 3.30 vs. 1.75, W=629.5, p = 0.0061). Total leukocytes, monocytes and thrombocytes were not different.

### 3.4 Diabetes-related associations with BM parameters

T1D was associated with a higher level of absolute CD34 counts (median 43.73 vs. 52.98, W=368, p= 0.0357) as well as %CD34 (median 0.57 vs. 0.94, W=424, p= 0.0171). However, given the small and imbalanced subgroup size, this finding should be interpreted as exploratory. Moreover, after adjustment for age and sex in the multivariable analysis, this association was no longer statistically significant.

**Figure 3:**
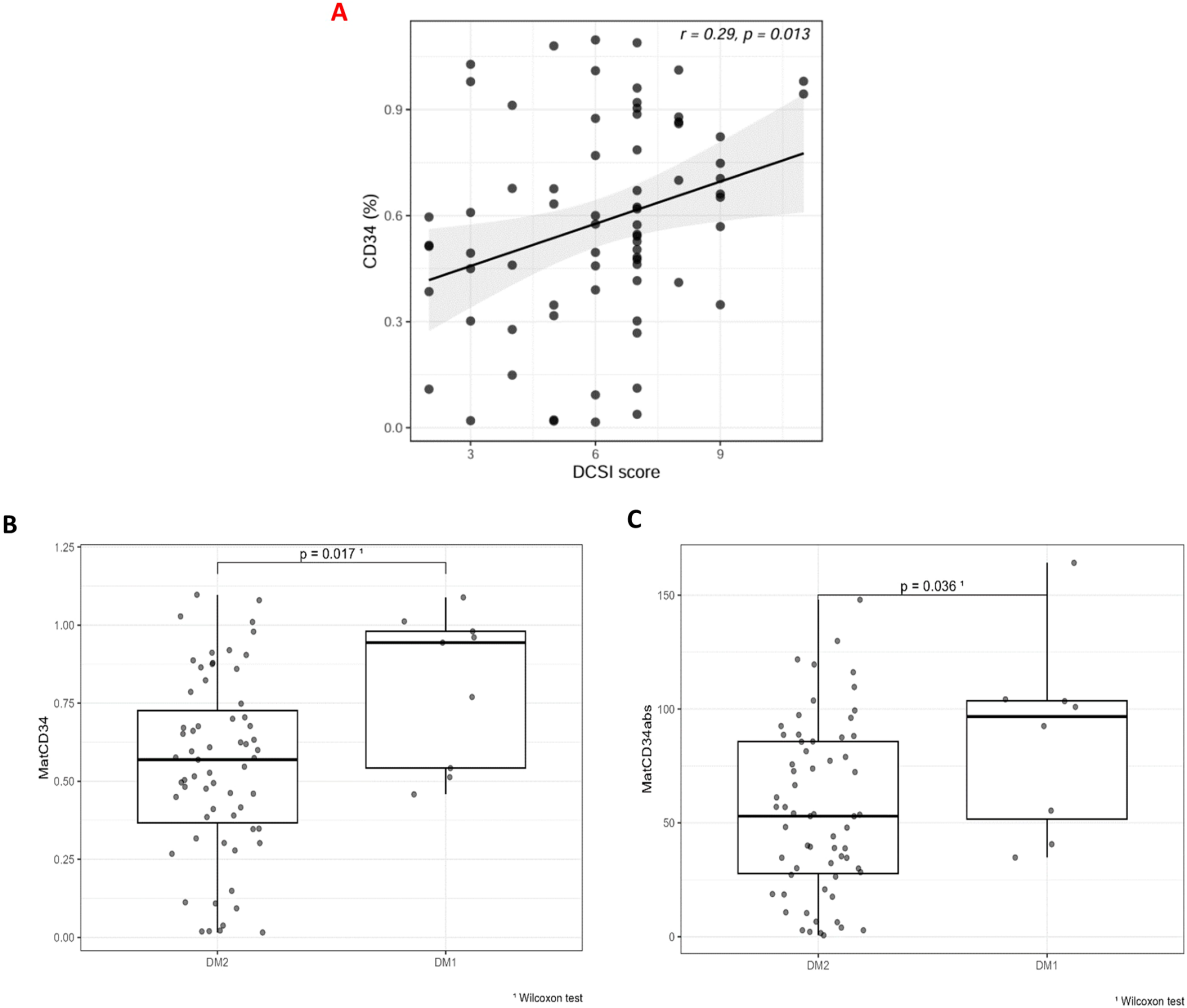
Graphs illustrating statistically significant associations between **(A)** CD34 cell percentage and diabetes complications severity index (DCSI) score, **(B)** CD34-positive cell percentage and diabetes type, and **(C)** absolute CD34-positive cell count (MatCD34abs) and diabetes type (DM1 = type 1 diabetes; DM2 = type 2 diabetes).

In patients with a higher score in DCSI classification %CD34 increased (β = 0.040, p= 0.013). No association was observed between the use of OADs or ACEI and BM parameters. The charts with significant results from this section are shown in **Figure 3**.

### 3.5 Key associations

The most important univariate associations between clinical predictors and bone marrow–derived cellular parameters are summarized in **Table 3**. Complete univariable analyses of all tested predictors are provided in the **Supplementary Material**-**Supplementary Table 1**.

**Table 3:** Summary of key associations between clinical predictors and bone marrow–derived cellular parameters.

Predictor	Outcome parameter	Estimate effect	P-value
Univariate analysis			
Statins	CD34%	median 0.67 vs. 0.43	<0.001
Statins	CD34 count	median 54.2 vs. 46.4	0.189
T1D	CD34 count	median 96.7 vs. 53.0	0.036
T1D	CD34%	median 0.94 vs. 0.57	0.017
DCSI	CD34%	β = 0.040	0.013
DCSI	CD34 count	β = 3.187	0.137
Multivariate analysis			
Statins	CD34%	β = 0.256	<0.001
T1D	CD34%	–	NS
DCSI	CD34%	–	NS
Additional analysis			
Lymphocytes	CD34%	median 1.75 vs. 3.3	0.006
Statins_men	CD34%	median 0.67 vs. 0.41	<0.001
Statins_T2D	CD34%	median 0.62 vs. 0.41	0.003

Multivariate analysis was performed only for variables meeting prespecified criteria in univariable analysis. T1D, type 1 diabetes; T2D, type 2 diabetes; CD34%, the percentage of CD34; DCSI, diabetes complications and severity index; NS, non-significant after adjustment.

## 4 Discussion

We found the use of statins to be associated with an increase in %CD34 without a significant increase in their absolute count, which leads us to hypothesize that statins influence the BM indirectly. Rosuvastatin has been shown to enhance the mobilization and migration of progenitor cells from the BM to circulation. These effects are mediated, at least in part, through activation of the eNOS–nitric oxide pathway and upregulation of chemokine signaling, particularly the CXCR4/SDF-1 axis, which are key regulators of progenitor cell trafficking and function. Such mechanisms may explain the observed statin-associated changes in BM composition without a concomitant increase in absolute CD34 cell counts (29).

An experimental study targeting prostate cancer demonstrated that statins — mainly atorvastatin and rosuvastatin — reduced BM adiposity in mice by suppressing the differentiation of BM stromal cells (BMSCs) into adipocytes *in vitro* and *in vivo*, which was associated with a decrease in adipocyte markers such as Adipoq, Plin, Fabp4, Lpl and Pparγ, as well as leptin levels (30). Another study suggests that simvastatin helps protect the BM niche after radiation partly through regulation of cytokine, including CXCL-12 (also known as SDF-1α), angiopoietin-1 (Ang-1), Jagged-1, and VEGF-A (vascular endothelial growth factor A), which are key regulators of hematopoietic stem cell (HSC) survival, proliferation, and engraftment, with increased expression promoting the maintenance and function of the marrow microenvironment after injury (31).

Moreover, another potential mechanism underlying the statin-associated increase in %CD34 is the reduction in lymphocyte counts, which may also explain the discrepancy between the increase in relative %CD34 and the unchanged absolute CD34-positive cell count. The reduction in lymphocyte counts observed during statin therapy may be related to the inhibitory effects of statins on lymphocyte proliferation and differentiation, particularly in T-lymphocyte and cytotoxic T-lymphocyte subsets. Proposed mechanisms include disruption of intracellular signaling pathways through inhibition of protein prenylation, increased apoptosis, and changes in gene expression that suppress lymphocyte development, activation, and function. In addition, statins may reduce lymphocyte migration, suppress pro-inflammatory immune responses, and alter adaptive immune homeostasis (31, 32).

Overall, statins promote BM function via activation of the eNOS-NO axis and increased VEGF signaling, which improves the vascular microenvironment and regenerative potential (33). These mechanisms include reduction of BM adiposity, protection of the hematopoietic niche, and modulation of cytokine signaling. This is supported by studies on their benefit in enhancing hematopoietic stem cell engraftment and expansion in transplantation and oncological therapy (14, 17, 30, 31, 34–36). Such an environment ensures a higher level of BM readiness to mobilize CD34 cells in response to ischemia, infection or pharmacological stimulation, which may be clinically useful, for mobilization before autologous transplantation and for ischemic tissue regeneration (37). Importantly, it has even been observed specifically in patients with no-option CLTI that atorvastatin therapy was associated with increased concentrations of bone marrow-derived mononuclear cells and improved transcutaneous oxygenation following ACT (15). An improved BM niche maintains progenitor cells in a quiescent state, preventing their depletion during chronic inflammation, anemia or aging - thereby contributing to the maintenance of long-term hematopoietic reserve (38). Our data therefore extend the prevailing model: in patients with diabetes and CLTI, statins do not necessarily mobilize additional CD34^+^ cells but rather sculpt a more progenitor-favorable microenvironment.

A more detailed description of BM characteristics is provided by the MLR. A higher MLR is associated with increased inflammation and immunosuppression in various diseases, reflecting a shift toward a myelomonocytic or immunosuppressive environment. However, MLR was not associated with statin therapy or other parameters (39).

In addition to the results discussed above, we found a positive association between both absolute count and %CD34 in patients with T1D. Several mechanisms explaining these results are described. The first process includes diabetic autonomic neuropathy (DAN), which may impact the BM by disrupting sympathetic regulation and altering key molecular pathways. Patients with T1D and associated DAN show a significant reduction in circulating CD34 cells by up to 40% compared to diabetic patients without DAN. This is especially explained by defective mobilization of hematopoietic stem and EPCs, which compromises vascular repair (4). Impaired mobilization results in the cells accumulating within the BM instead of being released into circulation (37). In contrast, T2D leads to greater BM fat infiltration, microangiopathy, and dysfunction of the BM niche, which further impairs CD34 cell regeneration. T1D tends to exhibit less marrow adiposity and consequently less stem cell depletion than T2D (5).

According to the DCSI scoring system, patients with higher complication burden have a higher %CD34. The most likely explanation is that in patients with advanced diabetes, the BM is often functionally depressed due to chronic inflammation, oxidative stress, and uremic toxins. CD34 cells may persist longer in the BM while the number of leukocytes and other mature elements decreases, as we have confirmed for lymphocytes, leading to a higher %CD34 despite unchanged absolute CD34 counts (37, 40). In advanced disease, fibrosis, hypoxia, and inflammation can damage hematopoietic niches, reducing cellular diversity and selective survival of early progenitors (5). The difference between the increase in %CD34 and stable CD34 count in patients with higher DCSI likely reflects BM remodeling and suppression, with preferential loss of mature cells, true stem cell expansion.

Importantly, most of the described alterations have been reported in the general population of patients with diabetes; however, our findings extend these observations to a highly selected subgroup of patients with diabetes and CLTI, in whom these changes may be more pronounced.

## 5 Limitations

This study has several limitations that may affect the interpretation of the results. The lack of morphological, histological, or functional evaluation of BM cells did not allow for the assessment of structural changes such as fibrosis, adipose tissue infiltration, or dysplasia. The cohort was predominantly composed of patients with T2D. This imbalance may have influenced the observed associations, reduced statistical power for subgroup analyses, and limited the generalizability of findings to patients with T1D. Consequently, analyses involving T1D should be considered exploratory and hypothesis-generating rather than confirmatory. A more balanced representation of both diabetes types would be necessary to allow more robust comparison of distinct pathophysiological mechanisms and BM alterations associated with T1D and T2D.

The absence of a control group without diabetes makes it difficult to distinguish diabetes-specific changes from those related to age or comorbidities. Glycemic control was assessed solely using HbA1c, which does not capture glycemic variability; the inclusion of continuous glucose monitoring would provide a more relevant assessment of glucose control. A selection bias was introduced by excluding patients with renal impairment, thus removing the principal component of the DCSI system and limiting extrapolation to the general population with an advanced DCSI score. We focused on medications with a documented relationship to BM properties. Other medications (e.g., antithrombotic therapy, anticoagulants, beta-blockers, diuretics, and others) were not analyzed due to the limited sample size. Additionally, no functional tests of hematopoietic cells were performed. Finally, the analysis was limited to CD34 cells, without additional surface markers that could distinguish between EPC, hematopoietic, and other progenitor cells. Extended immunophenotyping would allow deeper insight into the functional composition of the BM and its regenerative or immunomodulatory potential. Addressing these aspects in future research would greatly improve the mechanistic interpretation and translational relevance of BM changes in diabetes.

## 6 Conclusion

While BM alterations have been extensively described in patients with hematological disorders, our study is unique in that it focuses exclusively on patients with diabetes and limb ischemia who had no hematological indication for BM collection and no evidence of benign or malignant hematological disease. This study highlights the factors that affect BM in patients with diabetes and CLTI, especially the pleiotropic role of statins. Statins do not promote an increase in the number of CD34 cells, but modify cellular proportions in the BM, which could affect its hematopoietic function and BM niche. Their effect appears to support and protect the BM, which may be clinically relevant for transplantation purposes.

## Data Availability

The raw data supporting the conclusions of this article will be made available by the authors, without undue reservation.
