# Supplementary material for: Bone marrow cellular profile in patients with diabetes: association of statin therapy with CD34+ cells
Source: Front Endocrinol (Lausanne). 2026 May 29;17:1850519. doi: 10.3389/fendo.2026.1850519 (PMC13259772; doi:10.3389/fendo.2026.1850519)
Supplement: Supplementary file 1 [file Table1.docx]

**Table S1** Complete univariable analyses of clinical predictors and bone marrow–derived cellular parameters.

a. CD34 count, b. CD34 percentage c. amount of mononuclear cells d. monocyte-to-lymhocyte ratio.

DM – diabetes mellitus, HbA1c – glycated hemoglobin, DCSI – diabetes complications severity index, ACEI – angiotensin-converting-enzyme inhibitors, DmDuration – duration of diabetes, DPP4i – dipeptidyl peptidase-4 inhibitors, SGLT2i – sodium-glucose cotransporter 2 inhibitors, CKD – chronic kidney disease, NA – not applicable

1. **CD34 count**

| **predictor** | **estimate** | **group1_median** | **group2_median** | **statistic** | **p.value** | **method** |
| --- | --- | --- | --- | --- | --- | --- |
|  |  |  |  |  |  |  |
| DM | 43.728 | 52.983 | 96.711 | 368.000 | 0.036 | wilcox |
| HbA1c | 0.684 | NA | NA | 1.871 | 0.066 | linear |
| DCSI | 3.187 | NA | NA | 1.504 | 0.137 | linear |
| Statins | 7.781 | 46.421 | 54.202 | 474.000 | 0.189 | wilcox |
| non_HDL | 5.636 | NA | NA | 1.190 | 0.239 | linear |
| LDL | 4.758 | NA | NA | 0.833 | 0.408 | linear |
| Cholesterol | 3.729 | NA | NA | 0.774 | 0.442 | linear |
| Sex | 21.975 | 53.716 | 75.692 | 321.000 | 0.473 | wilcox |
| Metformin | 1.039 | 52.983 | 54.022 | 628.000 | 0.530 | wilcox |
| Insulin | -3.665 | 56.952 | 53.287 | 527.000 | 0.673 | wilcox |
| ACEI | 3.155 | NA | NA | 0.341 | 0.734 | linear |
| Age | -0.165 | NA | NA | -0.332 | 0.741 | linear |
| DmDuration | -0.108 | NA | NA | -0.303 | 0.763 | linear |
| DPP4i | -0.248 | 53.841 | 53.592 | 265.000 | 0.820 | wilcox |
| SGLT2i | -5.822 | 54.022 | 48.200 | 235.000 | 0.840 | wilcox |
| CKD | 0.928 | NA | NA | 0.162 | 0.871 | linear |

**b. CD34 percentage**

| **predictor** | **estimate** | **group1_median** | **group2_median** | **statistic** | **p.value** | **method** |
| --- | --- | --- | --- | --- | --- | --- |
|  |  |  |  |  |  |  |
| Statins | 0.233 | 0.433 | 0.666 | 293.000 | <0.001 | wilcox |
| Statins_men | 0.260 | 0.411 | 0.671 | 214.0 | <0.001 | wilcox |
| Statins_T2D | 0.205 | 0.414 | 0.619 | 308 | 0.003 | wilcox |
| Lymphocytes | -1.550 | 3.300 | 1.750 | 629.5 | 0.006 | wilcox |
| DCSI | 0.040 | NA | NA | 2.548 | 0.013 | linear |
| DM | 0.375 | 0.569 | 0.944 | 424.000 | 0.017 | wilcox |
| HbA1c | 0.005 | NA | NA | 1.838 | 0.071 | linear |
| Metforim | 0.096 | 0.504 | 0.600 | 679.500 | 0.279 | wilcox |
| CKD | 0.037 | NA | NA | 0.869 | 0.388 | linear |
| Age | -0.003 | NA | NA | -0.695 | 0.489 | linear |
| DmDuration | -0.001 | NA | NA | -0.412 | 0.682 | linear |
| DPP4i | 0.082 | 0.558 | 0.640 | 234.000 | 0.700 | wilcox |
| non_HDL | 0.010 | NA | NA | 0.289 | 0.773 | linear |
| Insulin | 0.007 | 0.569 | 0.576 | 480.500 | 0.774 | wilcox |
| SGLT2i | -0.094 | 0.576 | 0.482 | 238.000 | 0.849 | wilcox |
| LDL | -0.006 | NA | NA | -0.142 | 0.888 | linear |
| Sex | -0.030 | 0.575 | 0.545 | 302.000 | 0.903 | wilcox |
| Cholesterol | 0.004 | NA | NA | 0.106 | 0.916 | linear |
| ACEI | 0.002 | NA | NA | 0.034 | 0.973 | linear |

**c.** **Mononuclear cell count**

| **predictor** | **estimate** | **group1_median** | **group2_median** | **statistic** | **p.value** | **method** |
| --- | --- | --- | --- | --- | --- | --- |
| Statins | -1.295 | 3.45 | 2.155 | 778.500 | 0.081 | wilcox |
| Age | 0.046 | NA | NA | 1.549 | 0.126 | linear |
| SGLT2i | -0.470 | 2.49 | 2.020 | 343.000 | 0.171 | wilcox |
| Metformin | -0.755 | 2.91 | 2.155 | 515.000 | 0.218 | wilcox |
| Insulin | -0.310 | 2.52 | 2.210 | 610.500 | 0.278 | wilcox |
| CKD | 0.304 | NA | NA | 0.881 | 0.381 | linear |
| ACEI | -0.483 | NA | NA | -0.860 | 0.393 | linear |
| DM | -0.150 | 2.34 | 2.190 | 250.500 | 0.492 | wilcox |
| HbA1c | 0.014 | NA | NA | 0.602 | 0.549 | linear |
| DmDuration | -0.009 | NA | NA | -0.437 | 0.663 | linear |
| non_HDL | 0.127 | NA | NA | 0.426 | 0.672 | linear |
| DPP4i | -0.320 | 2.34 | 2.020 | 317.000 | 0.691 | wilcox |
| LDL | 0.118 | NA | NA | 0.343 | 0.733 | linear |
| Sex | 0.290 | 2.29 | 2.580 | 304.000 | 0.806 | wilcox |
| DCSI | -0.028 | NA | NA | -0.212 | 0.833 | linear |
| Cholesterol | 0.021 | NA | NA | 0.070 | 0.944 | linear |

**d. Monocyte-to-lymhocyte ratio**

| **predictor** | **estimate** | **group1_median** | **group2_median** | **statistic** | **p.value** | **method** |
| --- | --- | --- | --- | --- | --- | --- |
| Statins | 0.677 | -1.678 | -1.001 | 457.500 | 0.100 | wilcox |
| DCSI | 0.071 | NA | NA | 1.356 | 0.179 | linear |
| SGLT2i | 0.418 | -1.241 | -0.823 | 188.000 | 0.226 | wilcox |
| DM | 0.726 | -1.181 | -0.455 | 332.500 | 0.408 | wilcox |
| DmDuration | 0.006 | NA | NA | 0.668 | 0.507 | linear |
| Insulin | 0.423 | -1.469 | -1.046 | 452.000 | 0.514 | wilcox |
| Age | -0.007 | NA | NA | -0.608 | 0.545 | linear |
| Metformin | 0.289 | -1.360 | -1.071 | 649.000 | 0.553 | wilcox |
| DPP4i | -0.089 | -1.081 | -1.170 | 256.000 | 0.645 | wilcox |
| CKD | 0.056 | NA | NA | 0.404 | 0.687 | linear |
| Cholesterol | 0.030 | NA | NA | 0.257 | 0.798 | linear |
| ACEI | 0.057 | NA | NA | 0.254 | 0.800 | linear |
| Sex | -0.276 | -1.071 | -1.347 | 326.000 | 0.800 | wilcox |
| LDL | 0.028 | NA | NA | 0.208 | 0.836 | linear |
| non_HDL | 0.023 | NA | NA | 0.201 | 0.842 | linear |
| HbA1c | 0.001 | NA | NA | 0.079 | 0.937 | linear |
